# Mental Health Clinician Attitudes about Service User and Family Agency and Involvement in Recovery-Oriented Practice

**DOI:** 10.3390/ijerph20186787

**Published:** 2023-09-20

**Authors:** Janice Chisholm, Judy Hope, Ellie Fossey, Melissa Petrakis

**Affiliations:** 1 Department of Social Work, Faculty of Medicine, Nursing and Health Sciences, Monash University, Melbourne, VIC 3145, Australia; 2 Mental Health and Wellbeing Program, Eastern Health, Centre for Education and Research, Melbourne, VIC 3128, Australia; 3 Eastern Health Clinical School, Faculty of Medicine, Nursing and Health Sciences, Monash University, Melbourne, VIC 3128, Australia; 4 Department of Occupational Therapy, Faculty of Medicine, Nursing and Health Sciences, Monash University, Melbourne, VIC 3199, Australia; 5 St Vincent’s Hospital Mental Health Service, Melbourne, VIC 3065, Australia

**Keywords:** recovery-oriented practice, mental health, lived experience, families, carers, multidisciplinary clinicians, biomedical model

## Abstract

Background: Recovery-oriented practice (ROP) is a framework focusing on recovery through hope, choice, and meaning, to live with or without enduring symptoms and challenges. Aims: To examine clinicians’ attitudes about the involvement of service users and family or supporters in ROP. Methods: A bespoke Qualtrics survey obtained views of mental health clinicians working in an Australian public mental health service about service user and family involvement in ROP, using a five-point Likert scale of agreement and free-text responses. Data were analysed with descriptive statistics and content analysis methods. Results: Two hundred and three clinicians completed the survey. Most (79%) clinicians agreed with the statement that service users want clinicians to use ROP principles, and the majority (63%) also ‘strongly believed’ that ROP made a difference to service users’ mental health outcomes. Only 15% ‘strongly agreed’ and 57% somewhat agreed with the statement that service users know what treatment is best for them, and only 20% of clinicians ‘strongly agreed’ that supporters of service users believed in and wanted ROP for their family member or friend. Future directions: This study adds to the literature on clinicians’ views about ROP and shows that although clinicians are supportive of ROP, they also express substantial ambivalence about whether service users and families know what treatment is best. For ROP implementation to be successful, workforce training needs to support clinicians to reflect on these views with service users and families, and to encourage supported decision making. Future studies should focus on changes in clinicians’ views and practice post ROP training.

## 1. Introduction

Recovery-oriented practice (ROP) in mental health has become a global movement in the delivery of mental health services and there have been many studies describing how it can be implemented [1,2,3,4]. Recovery-oriented practice evolved out of critique from human rights and service user movements challenging thinking about mental illness and mental health practice [5,6,7]. A well-accepted definition of recovery is found in Anthony [8] (p. 15);

“Recovery is a deeply personal, unique process of changing one’s attitudes, values, feelings, goals, skills, and/or roles. It is a way of living a satisfying, hopeful, and contributing life even within the limitations caused by illness. Recovery involves the development of new meaning and purpose in one’s life as one grows beyond the catastrophic effects of mental illness”.

Recovery-oriented practice (ROP) therefore aspires to change how mental health services are delivered, and become foundational to mental health care that is service-user-driven, values and respects service users’ views and experiences, maximises service users’ self-determination, and supports their strengths and possibilities for recovery [9,10,11,12].

Over the last couple of decades, investigations of ROP have demonstrated it to be supportive of the different pathways to the recovery of service users [13,14,15,16]. Service users report wanting authentic engagement; to be treated with respect and dignity; and for language to be used that is inclusive and not “othering” to them [17,18,19,20]. The lived experience perspectives of service users, supporters and peer workers are primary in ROP [18,21]; however, clinicians’ understandings of and attitudes toward ROP are also important as clinicians hold powerful positions in mental health services. Clinician attitudes and commitment to ROP are essential to its successful implementation in day-to-day service delivery with service users and families.

Some evidence of clinician views of ROP has been emerging. Some international studies have focused on whether clinicians correctly identify ROP practice. For instance, studies in the UK and Australia have reported that ROP may erroneously be seen by clinicians as something that they engage in, rather than a shared experience between service user and clinician, or clinicians may erroneously believe they are already practicing ROP [22,23]. A recent South African study also reported clinicians had little idea about ROP and favoured a medical model of practice, but expressed the desire to train in ROP [24].

Clinicians’ attitudes towards ROP and service users have shown mixed results. An Australian study in an inpatient rehabilitation setting described progress towards ROP, including that clinicians focused on imparting hope for service users’ future dreams, providing personalised care, and valued their more equal involvement in decision making [25]. Similarly, in an acute inpatient mental health service in New Zealand, nurses described their practices in promoting safety, meaningful engagement, and their collaboration with service users as recovery-oriented [22]. In a larger scale implementation of ROP through the training of multidisciplinary community and rehabilitation teams in the United Kingdom [25], the role of hope for service users and nurses alike, and the importance of the language of recovery were also highlighted. Nurse clinicians in this study identified medications and symptom management as primary to treatment, although they also understood that ROP is more than this [23]. In comparison, a Danish ethnographic study in (2019) [(26)] described a range of approaches adopted by clinicians that were less consistent with ROP. For instance, when choosing life goals and expressing preferences in terms of treatment planning did not easily come to service users, clinicians in this study took control of this or negotiated contingency-based agreements with service users; for example, service users could have something if they took a certain medication [26]. Thus, in this inpatient setting, it appeared that ROP principles were only adhered to when it suited clinicians’ workload or timing; in other words, ROP was seen as ancillary [26]. Other barriers to the implementation of ROP reported by clinicians include competing clinical and recovery orientations [27], the hierarchical nature of organisations, resourcing and time issues, and impracticable expectations in their work settings [27].

Recovery-oriented training packages can make a difference to clinician uptake of ROP [28], change clinician skills, knowledge and attitudes towards the ROP framework [29], and improve service user experiences of mental health care [30]). Despite the breadth of knowledge about clinician attitudes to ROP in inpatient and community practice settings, less is known about clinician views regarding service user and family involvement in ROP. While service user and family views are central to implementing ROP, this study focused on clinician views. The justification for this lays in “theory of mind”, which allows for the respondents to predict others’ intentions and understand their own state of attribution [31]. In this way, clinicians’ understandings of service users and families can be known in order to assist with what may be required for the future training of clinicians. This “theory of mind” perspective (i.e., one person’s view of another person’s perspective) was chosen since it may provide useful information to guide workforce development initiatives aimed at supporting the effective implementation of ROP by clinicians in mental health services. Therefore, this study addressed clinician views about ROP practice and impact, and explored their views on whether service users and families want ROP to be implemented.

## 2. Study Aims

The present research aimed to examine clinicians’ attitudes about implementing ROP principles within their practice with service users and their supporters. It specifically examined the research questions:

Do clinicians believe service users want recovery principles used in the clinician–service user interaction?

Do clinicians believe using ROP makes a difference to service users’ mental health outcomes?

Do clinicians believe service users can decide what treatment is best for them?

Do clinicians believe families and carers support an ROP framework and want it for their family member?

## 3. Material and Methods

### 3.1. Study Setting

The research was undertaken in a public mental health service in an urban area in the southern state of Victoria, Australia. While mostly urban, this mental health service extends across 3000 square kilometres, includes some semi-rural areas, and services over 750,000 people across the lifespan. It is multidisciplinary and employs over 700 mental health clinicians.

### 3.2. Research Design

The present study sits within a broader exploratory, narrative, and lived-experience-inclusive project. An initial study explored and reported the views of lived experience staff including service user and carer consultants and peer workers [18,21]. A second project described the views of clinicians about working alongside peer workers and clinicians’ sense of efficacy about implementing ROP [21]. The present research sought the views of multidisciplinary clinicians including social workers, occupational therapists, nurses, psychologists, psychiatrists, and medical staff about service user and family/supporter involvement in ROP.

### 3.3. Survey Instrument and Data Collection

Purposive sampling was utilised to recruit staff across the whole public mental health service. Purposive sampling was chosen because this would reach the respondents desired in the sample, i.e., staff with experience working in one or more of the mental health teams within operating in this service. The survey was a purpose-built, targeted, and bespoke Qualtrics survey designed to obtain clinicians’ views about ROP and having family members and supporters involved in service users’ recovery, and whether service users could choose what treatment was best for them. The four items included in the present paper are those in relation to clinician self-reported opinions about service user and family experiences with ROP, and service users’ beliefs and preferences regarding treatment. Demographic details were collected, which included discipline, team, hours of work, and length of experience. Clinician opinions were collected using a five-point Likert scale of agreement. There were options for free-text responses. The voluntary survey was disseminated via email to all mental health staff at the study service; 12 weeks were given to complete the survey with four weekly reminders, and then a final two-week reminder. Ethics approval was granted by the study service (Reference number: QA48-2016) and associated university (review reference number: 2016-0575-341) prior to data collection.

### 3.4. Data Analysis

Descriptive statistics were generated using SPSS version 16. Data were organised and presented graphically and/or described where indicated. The design of the survey and the analysis of survey data followed that of the Consensus-Based checklist for Reporting of Survey Studies (CROSS), making it reliable, clear, and easily reproduced [32].

Content analysis is an adaptable way of analysing text data [33], which can be used for open-ended qualitative survey responses to identify patterns in the text through subjective interpretation [34]. Hsieh and Shannon describe three different approaches to content analysis: conventional, directed, and summative. In this study, a summative content analysis was used because the qualitative data yield was small.

## 4. Results

This paper builds on previous research completed by the authors focused on peer workers’ [18,35] and clinicians’ [21,22] perspectives about other aspects of ROP and its potential for implementation at the study service.

### 4.1. Demographic Information

Participants were asked a qualifying question about whether they worked in mental health at the study service. A total of 203 clinicians commenced and completed the survey (around 27% response rate). There were 142 females and 46 males. Fifteen participants did not record their sex. Participants included 48% full-time, 37% part-time, and 5% were casual clinicians.

As shown in Table 1, the clinicians worked in various multidisciplinary mental health settings with representation of participants from all teams in the study service. Over half of the participants worked in community services including adult, youth, and aged services. Nearly one quarter of respondents worked in acute settings.

Table 2 shows the professional discipline, number of clinicians, and median years of employment.

As shown in Table 2, nearly half of all the participants were registered nurses; however, the numbers of participants from the different professional groups were similar in proportion to those employed in the service. Over one third of them had worked in mental health for over 20 years, with an average of 14 years of experience.

There was no bias in professional discipline in the responses to the items that follow, having calculated each professional discipline with the items in this study.

Table 3 describes the items and results from the Likert scale responses. The responses are in percentages and numbers of participants.

Item 1 shows that 79% of clinicians believed service users wanted them to work in a recovery-oriented way, whereas 14% of clinicians indicated service users did not want ROP principles utilised. They were relatively equal in terms of the discipline in reporting in favour of this statement.

Item 2 shows that a high percentage (82%) of clinicians disagreed with the statement indicating strong support for ROP making a difference to service users. Still, there were just over 10% who responded contrarily. They were relatively equal in terms of the discipline in reporting in favour of this statement.

Item 3 shows that the majority agreed with the statement “I believe service users can decide what treatment is best for them” (71%). However, 28% of respondents disagreed. Over half of the psychiatrists and one third of registered nurses disagreed with this statement. Those who disagreed worked variously in acute and community services and had varying lengths of experience working in mental health. Of those that disagreed, 8% ‘strongly disagreed’.

Item 4 shows overall agreement (73%) with the statement “families and carers support recovery-oriented practice and want it for their family member”. However, 25% of clinicians disagreed, of whom two thirds were registered nurses, one in five were psychiatrists, and one quarter were allied health staff.

Table 4 describes free-text responses to the item “I believe service users can decide what treatment is best for them”. There were no qualitative responses to the other items.

### 4.2. Content Analysis

Each respondent to the statement, “I believe service users can decide what treatment is best for them” answered “agree somewhat”, and then appeared to qualify this response using free text. The first and fourth respondents were nurses of different levels of experience, who indicated that agreement with treatment was more important than agency or choice. These responses used paternalistic language, including the idea of “containment/protection”, to keep service users “safe” in the event of relapse. The second and third respondents gave more tempered responses and relied more on the individual circumstances of the service user and the value of forward planning to uphold choice. Nonetheless, both respondents appeared to shrink back from their agreement about service users being able to determine their own treatment. The third and fourth respondents noted “involuntary treatment” or “treatment orders” in particular as the basis for their reservations about this statement.

## 5. Discussion

This study aimed to better understand mental health clinicians’ attitudes toward service user and family/supporter involvement in ROP. The overall results highlight that most of the surveyed mental health clinicians thought service users wanted them to practice in a recovery-oriented manner, and that doing so made a difference to service users’ mental health outcomes. In terms of the qualitative data, emergent future-focused allyship was evident; however, most comments still contained paternalistic foundations.

### 5.1. Clinicians’ Thoughts Regarding Engaging Service Users in Recovery Principles

Most clinicians (79%) reported that service users did indeed want them to utilise ROP in their work. Over 80% reported that ROP made a difference to service users’ mental health outcomes. This aligns with findings from [33] in which over 80% of clinicians reported that they actually utilised ROP principles in their work with service users.

The views expressed by the majority of survey respondents were consistent with the well-established understanding that service users want to be engaged in their recovery and want effective communication and respect from clinicians to achieve their recovery goals [36,37]. Omeni and Barnes [38] demonstrated that both service users and clinicians desire greater input from service users in the decision-making process. Clinicians believed the advantages of service user involvement to include: being informants of knowledge, feelings of empowerment and self-esteem for service users, and professional development for clinicians [38]. The negative aspects that clinicians outlined were that service users were too unconstructive, critical, tokenistic, and not representative of all service users, and they were not important [38,39]. Similarly, Chisholm and Petrakis [32] outlined clinicians’ commitment to and understanding of ROP even prior to formal training. Clinicians did not think it would necessarily be easy to implement ROP service-wide but believed culture and service provision change could occur.

In the present study, the majority of clinicians believed service users wanted ROP principles used, although 15% stated otherwise. Chisholm and Petrakis [22] found that clinicians knew what ROP was, felt efficacious in providing it, and were keen to implement it into their practice. Clinicians in other studies also held largely optimistic views and adopted recovery principles for work with service users [40].

In the current study, some clinicians held unhelpful views about ROP and services. Negative staff attitudes toward service users could indicate that there is a need for further training and support to implement recovery-oriented practices [41]. These attitudes were not found to a large extent in the Chisholm and Petrakis [22] study within the same service setting, in which clinicians reported supporting ROP and its implementation. Hansson and Jormfeldt [41] stated that evidence-based practices may assist in improving clinicians’ attitudes toward ROP and service users.

Negative and stigmatising attitudes held by clinicians can make therapeutic work difficult [40,42] and can add to clinician burnout [40]. This could make utilising ROP principles difficult with service users. The present study found that clinicians thought service users wanted ROP and that it was beneficial to them. This indicates that the clinicians in the present study held largely positive attitudes about ROP with service users. Since stigmatising attitudes among clinicians have been found to be detrimental to good recovery outcomes for service users [43], training and staff monitoring in this area may be indicated in the current setting and particularly in service settings embarking the transition to ROP.

### 5.2. Clinicians’ Beliefs Regarding Service Users’ Decision Making about Treatment

Nearly one third (30%) of clinicians did not think service users could choose the best treatment for themselves. Further, the majority of clinicians were ambivalent about service users being able to make decisions about their treatment, and the text responses reflect this ambivalence. Most of these views (75%) were from psychiatrists and registered nurses who completed the survey, although a quarter were from social workers, and all the text responses reflected staff being sceptical of service users being able to choose options rather than be treated with coercive treatment. This result is perhaps unsurprising, given that the study included inpatient units and community services where community treatment orders were used, both being contexts in which implementing ROP is reported as challenging [44]). These together suggest that some clinicians may find themselves caught in the middle between practicing in a recovery-oriented way whilst practicing within a clinically oriented system that endorses coercion associated with the biomedical approach [36]. There were some clinicians who highlighted that when a service user is deemed unable to consent, it is difficult to implement some of these principles, despite the known principle that service users should be consulted as much as possible [45].

Clinicians have various views about service users that may rationalise their experiences with ROP. Clinicians have been found to believe that they are collaborative, despite their use of coercive treatment [46], and report a high degree of support for the use of restraints even when service users are not being a risk to themselves or others [46]. Strategies to improve attitudes toward autonomous decision making may include reflective practice, supported decision-making practices, and the use of advance statements of preference. This suggests that the inclusion of these areas in ROP training may help clinicians to shift practice toward considering what resources and supports may uphold a service user’s right to stay in control of their decision making [30].

### 5.3. Clinicians’ Thoughts Regarding Families and Carers Views of Recovery-Oriented Practice

The majority (73%) of clinicians thought “families and carers support recovery-oriented practice and want it for their family member”, while only 20% ‘strongly agreed’, and 25% did not agree at all. This result may be related to clinicians’ pessimism regarding treatment choices by service users. Little has been written about this phenomenon, which bears further investigation.

Clinicians in the present study may have also reflected that supporters, families, and carers are afraid that their concerns will not be included in their loved one’s recovery, despite ROP having connectedness and participation as components. Conversely, Giacco et al. [45], in their paper on carer involvement, found clinicians to be favourable towards carer involvement, especially with positive concerns for ROP principles. Families welcomed involvement that did not leave them marginalised and their opinions disregarded, and when they learned more about ROP principles, they were more hopeful and engaged with social workers [47]. Although Wyder and Bland [48] identified social workers as well-placed to support this work, recovery-oriented practice ideally supports the inclusion of carer and service user consultants and peer workers in mental health services to connect with families and use of ROP principles. There is a need for training to include a greater emphasis on transdisciplinary working in relation to ROP, highlighting shared principles, behaviours, and messages under this approach, to ensure that the broad staff team adopt the practices.

### 5.4. Strengths and Limitations

This study describes a single organisational workforce at a single point in time. The direct generalisability of the findings, to the broader service sector and international practice contexts, may therefore be limited. The study has other potential limitations: the survey was conducted using a non-standardised tool with Likert scales and free-text response options, and there may be response bias in the sampling method. The strengths of the study include the substantial proportion of the workforce who responded to the survey. Although the survey response was strong (27%), the non-responders may have been less informed and interested in ROP. However, the response rate to the survey pleasingly included all disciplines and broadly representative demographics. While it is not possible to make fully accurate inferences about the 73% of clinicians who did not respond to the survey, it might be expected that further opportunities for recovery-oriented training would enhance their skills and knowledge for implementing ROP. Understanding clinicians’ views about these topics is vital for the successful implementation of ROP, and for moving clinical practice away from the dominant biomedical model. Practically, it will be important to investigate this in light of clinicians working more and more closely with lived experience peer workers.

### 5.5. Future Research

Future research will be needed within the study service to ascertain the impact of ROP over time on staff attitudes, behaviours, and orientation to embracing recovery as the preferred and typical outcome in the lives of service users, rather than the exception. Nationally and internationally—since ROP is both evidence-informed and supported by service users, as well as valued by them—there is a need for future studies to investigate uptake, facilitators, barriers, and processes for implementation. Such research should include specific studies regarding the tailoring of ROP initiatives to meet the nuanced needs of priority populations of people who may have experienced additional barriers to wellbeing, due to intersectionality and the multiplied stigma that can bring, including people who identify as LGBTIQ+, multicultural and multi-faith communities, and people in under-resourced rural and remote communities. There is a need for training to include a greater emphasis on transdisciplinary working in relation to ROP, highlighting shared principles, behaviours, and messages under this approach, to ensure that the broad staff team adopt the practices.

## 6. Conclusions

This study showed that most clinician respondents stated that they believed ROP was the desired model to support service users in their recovery. Areas where more change is possible were attitudes toward autonomy in treatment decision making and beliefs about a family’s support of ROP, which warrant further exploration. The present study adds to the growing available literature on ROP implementation, and highlights areas potentially requiring workforce training and support for reflective practice. Strategies such as employing service user and family peer workers in the clinical space may enhance the implementation of ROP principles by clinicians through deeper cultural shifts towards respect, collaboration, and co-learning, rather than the perpetuation of power differences in care relationships.

## Figures and Tables

**Table 1:** Mental Health Teams.

Team	Number	Percentage
ACCESS/Triage	19	9%
Community Care Unit (CCU)	10	5%
Child Youth Mental Health Service (CYMHS)	22	11%
CYMHS Intensive Monitoring Team (IMT)	4	2%
Adult Crisis Assessment & Treatment Team (CATT)	7	3%
Adult Mental Health Service—Continuing Care Team (CCT) and Inpatient Unit (IPU)	92	47%
Adult Mental Health Service Mobile Support & Treatment Service (MSTS)	9	4%
Aged Person’s Mental Health Service—Community and Inpatient Unit (IPU)	15	7%
Spectrum—specialist service for persons with a personality disorder	10	5%
Other—Longer term recovery support programs	15	7%
Total	203	100%

**Table 2:** Professional Discipline.

Professional Discipline	Number	%	Median Years Working
Registered Nurse	96	47%	10–15
Social Worker	26	13%	5–10
Occupational Therapist	21	10%	10–15
Psychologist	18	9%	10–15
Other	16	8%	5–10
Psychiatric Registrars	14	7%	2–5
Psychiatrists	8	4%	15–20
Enrolled Nurse	4	2%	15–20
Total	203	100%	10–15

**Table 3:** Results of Likert Scale Items.

Item	Disagree Strongly % & No.	Disagree Somewhat % & No.	Don’t Know % & No.	Agree Somewhat % & No.	Agree Strongly % & No.
Service users are not interested in me using recovery principles in my practice with them	47% (95)	32% (65)	6% (12)	14% (28)	5% (3)
2. Recovery-oriented practice does not make a difference to service users’ mental health outcomes	62% (126)	20% (42)	6% (12)	10% (21)	<5% (2)
3. I believe service users can decide what treatment is best for them	8% (16)	20% (42)	3% (4)	58% (118)	13% (27)
4. Families and carers support recovery-oriented practice and want it for their family member	2% (4)	24% (49)	1% (2)	53% (107)	20% (41)

**Table 4:** Qualitative responses.

Professional Discipline	Years of Experience	Location	Rating to Question	Qualitative Response to ‘I Believe Service Users Can Decide What Treatment is Best for Them’
Registered Nurse	2–5 years	Adult Inpatient Unit	“agree somewhat”	“Sometimes consumers may need medications but don’t agree.”
Social Worker	15–20 years	Adult Inpatient Unit	“agree somewhat”	“Clients should have an active Recovery plan which includes their preference for medication, treatment options (e.g., IMI, oral, depo, ECT) and restraint/seclusion or time out.”
Registered Nurse	5–10 years	Adult Inpatient Unit	“agree somewhat”	“I don’t think there is a blanket answer to any of the questions just as I believe that there are instances where a consumers life is of higher quality as a result of involuntary treatment. Each case must be judged on its individual merits.”
Registered Nurse	10–15 years	Adult Community Continuing Care Team	“agree somewhat”	“Though my responses seem positive, I do have reservations with recovery orientated practice in the area of discharging clients from treatment orders either too soon whether due to limited insight with client and potential for them to relapse. I feel that in the long term we may be dealing with more admissions and relapses due to reduced containment/protection of treatment orders.”

## Data Availability

Please contact the corresponding author.
